# A High-Quality Genome Sequence of Model Legume *Lotus japonicus* (MG-20) Provides Insights into the Evolution of Root Nodule Symbiosis

**DOI:** 10.3390/genes11050483

**Published:** 2020-04-29

**Authors:** Haoxing Li, Fan Jiang, Ping Wu, Ke Wang, Yangrong Cao

**Affiliations:** 1 State Key Laboratory of Agricultural Microbiology, College of Life Science and Technology, Huazhong Agricultural University, Wuhan 430070, China; haoxing_li@126.com (H.L.); wupingjob@163.com (P.W.); cocowant@126.com (K.W.); 2 College of Informatics, Huazhong Agricultural University, Wuhan 430070, China; fjiang@webmail.hzau.edu.cn

**Keywords:** *Lotus japonicus*, genome, Hi-C assembly, assembly evaluation, phylogenetic

## Abstract

*Lotus japonicus* is an important model legume for studying symbiotic nitrogen fixation as well as plant development. A genomic sequence of *L. japonicus* (MG20) has been available for more than ten years. However, the low quality of the genome limits its application in functional genomic studies. Therefore, it is necessary to assemble high-quality chromosome sequences of *L. japonicus* using new sequencing technology to facilitate the study of functional genomics. In this report, we used the third-generation sequencing combined with the Illumina HiSeq platform to sequence the genome of *L. japonicus* (MG20). We obtained 544 Mb of genomic sequence using third-generation assembly. Based on sequence analysis, 357 Mb of repeats, 28,251 genes, 626 tRNAs, 1409 rRNAs, and 1233 pseudogenes were predicted in the genome. A total of 27,991 genes were annotated into databases. Compared to the previously published data, the new genome database contains complete *L. japonicus* sequences in the proper order and orientation with a contig N50 2.81Mb and an excellent genome coverage, which provides more accurate genome information and more precise assembly for functional genomic study.

## 1. Introduction

*Lotus japonicus* (MG-20), an important model plant of the Leguminosae, is a wild early-flowering accession collected on Miyako Island, the southernmost point of the Japanese archipelago, which was later named as Miyakojima MG-20 [1]. The *L. japonicus* (MG-20) is a diploid (2*n* = 12) with six chromosomes and 472.1 Mbp genome size determined on the condensation patterns and the locations of rDNA loci [2]. *L. japonicus* is widely used by researchers worldwide to study the molecular mechanisms related to symbiotic interactions with rhizobia as well as plant development. Several essential components involved in the nodulation signaling pathway have been cloned and functionally characterized via forward genetics in *L. japonicus*, including Nod Factor Receptors 1 and 5 (NFR1 and NFR5), Symbiosis Receptor Kinase (SYMRK), NSP1, etc. Genome-wide research not only helps to reveal the relationship between genetics and evolution, but also to discover some new genes with unknown functions: for example, new genes related to phosphate accumulation [3], as well as genes related to the phenotypic changes in overwintering and flowering time due to regional differences [4].

A previously published genome assembly of *L. japonicus* (MG-20) based on clone-by-clone sequencing and shotgun sequencing technologies contains 394 Mbp with 44,464 sequence contigs [5]. Gifu and Miyakojima MG-20 are two ecotypes of *L. japonicu*s with high accuracy in their genomes of 494MB and 512MB, respectively [6]. The nucleotide sequence of the entire chloroplast genome (150,519 bp) of a legume, *L. japonicus* (MG-20), has also been determined [7]. Previous studies have used FISH analyses to correlate genetic maps and chromosome maps [2,8]. However, the low quality of this genomic sequence limits its application in functional genomic studies. Here, we performed high-depth (more than 60×) sequencing of *L. japonicus* (MG20) using the Illumina HiSeq 2500 platform and PacBio sequencing system and obtained total 43.57 Gb of data, with the number of reads at 4,671,067 and the mean read length at 9328bp. The data from Hi-C generated a highly contiguous reference genome of 499 Mb with 616 sequence contigs. We constructed an evolutionary tree based on single-copy genes from the genomes of 10 species and calculated the approximate divergence time of *L. japonicus* using r8s, which is a software package used to infer divergence times on a molecular phylogeny using penalized likelihood, maximum likelihood, and nonparametric rate smoothing methods. The new fully assembled genome was named *LjPB_ver1.0* in order to distinguish it from the description of the previous version.

## 2. Materials and Methods

### 2.1. Genome Sequencing

Fresh young leaves were collected from a single *L. japonicus* plant and immediately frozen in liquid nitrogen. Genomic DNA was extracted from the sample using the CTAB method. The DNA was purified and fragmented into 300–700 bp fragments, and the interacting DNA fragments were captured by streptavidin magnetic beads for library construction. High-throughput sequencing was performed using the Illumina HiSeq 2500 system.

After filtering out linker sequences, low-quality reads, and short reads (<500 bp), 43.57 Gb of sequencing data were obtained. The mean read length was 9328 bp. Canu [9] is a fork of the Celera Assembler designed for high-noise single-molecule sequencing (available at https://github.com/marbl/canu, v1.5). In the correction step, Canu selects longer seed reads with the settings ‘genomeSize = 430000000’ and ‘corOutCoverage = 0’ and then detects overlapping raw reads using the highly sensitive overlapper MHAP (mhap-2.1.2, option ‘corMhapSensitivity =low/normal/high’). Canu then performs error correction using the falcon_sense method (option ‘correctedErrorRate = 0.025’). Next, using default parameters, the error-corrected reads are trimmed of unsupported bases and hairpin adapters to obtain the longest supported range. Finally, Canu generates a draft assembly using the 80 trimmed reads with the longest coverage.

Falcon [10] is a hierarchical and haplotype-aware genome assembler (available at https://github.com/PacificBiosciences/FALCON, v0.3.0). In the correction step, Falcon selects longer seed reads with the setting ‘length_cutoff = 3000’ and then detects overlapping raw reads with daligner overlapper (pa_HPCdaligner_option, ‘-v -B128 -e.70 -l4800 -s100-k18 -w8 -h480 -M8 -T4’). Falcon then performs an error correction step using the falcon_sense method (falcon_sense_option, ‘--output_multi --min_idt 0.70 --min_cov 3 --max_n_read 200 --n_core 4’). In the assembly step, Falcon selects pre-assembly reads with the setting ‘length_cutoff_pr = 8000’, detects overlapping reads (ovlp_HPCdaligner_option, ‘-v -B128 -e.96 -l2400 -s100 -k18 -h1024 -M8 -T4’), and constructs a directed string graph with the setting ‘overlap_filtering_setting = --max_diff 120 --max_cov 120 --min_cov 3 --n_core 4 --bestn 8’. Finally, correction by Pilon software [11] was used to correct the assembly based on second-generation data.

### 2.2. Hi-C Assembly

We constructed Hi-C fragment libraries with insert sizes ranging from 300–700 bp as described in Rao et al. [12] and sequenced the libraries using the Illumina platform. Briefly, adapter sequences were trimmed from the raw reads and lower-quality PE reads were removed to obtain clean data. The clean Hi-C reads, accounting for 60× fold coverage of the *Lotus japonicus* genome, were truncated at the putative Hi-C junctions, and the resulting trimmed reads were aligned to the assembly using bwa aligner [13]. Only uniquely aligned read pairs whose mapping quality was >20 were retained for further analysis. Invalid read pairs, including dangling-end and self-cycle, re-ligation and dumped products, were filtered out using HiC-Prov2.8.1 [14]. Of the uniquely mapped read pairs, 79.56% were valid interaction pairs and were clustered, ordered, and oriented into scaffolds and onto chromosomes with LACHESIS [15] (option, ‘CLUSTER_MIN_RE_SITES = 22’, ‘CLUSTER_MAX_LINK_DENSITY = 2’, ‘CLUSTER_NONINFORMATIVE_RATIO = 2’, ‘ORDER_MIN_N_RES_IN_TRUN = 10’, ‘ORDER_MIN_N_RES_IN_SHREDS = 10’).

### 2.3. Evaluation of the Assembly

The assembly results were evaluated using three methods: (1) the assembly was compared to the second-generation sequencing data; (2) the single-base error rate was calculated; and (3) the genome integrity was assessed. To compare the assembly to the second-generation sequencing data, clean reads were used to create clean read data files, with one unit per four rows and double-ended statistics; for example, read1 and read2 were recorded as two reads. Mapped reads (%), indicating the number of clean reads targeted to the reference genome divided by the percentage of all clean reads, were determined using the samtools flagstat command. Properly mapped (%) double-end sequences were mapped to the reference genome, and the distance was consistent with the length distribution of the sequencing fragments, as determined using the samtools flagstat command. To evaluate single-base error rate, the second-generation sequencing reads were compared to the assembled genomic sequences, and the single-base error rate of the genome was estimated by determining the ratio of the number of bases in the genome that were inconsistent with the sequencing reads.

Genome integrity was assessed using BUSCO software. The embryophyta_odb9 database in BUSCO v2 [16] contains 1440 conserved core genes in terrestrial plants. We used BUSCO v2.0 software to assess the integrity of the Lotus genome assembly. CEGMA v2.5 [17] contains 458 conserved core genes in eukaryotes. We also used CEGMA v2.5 to assess the integrity of the genome assembly. For Hi-C library quality assessment, we aligned the sequencing data with the sequences of the assembled genomes using BWA [18]. An excessively long insert implies that the fragment may be formed by random joins. Therefore, read pairs that were larger than the maximum insert length were filtered out. To evaluate the Hi-C data based on invalid interaction pairs and valid interaction pairs, the genome sequence was divided, sorted, and oriented using LACHESIS software, and the assembly results were evaluated. For genomic error correction of the assembly, interrupting contigs were identified based on a length of 300 kb. The contigs were reassembled with Hi-C, and the positions that could not be restored to the original assembly sequence were listed as the candidate error area. The position of the low Hi-C coverage depth in this area was identified as the error point, thus completing the correction of the preliminarily assembled genome. The error-corrected genome was assembled using LACHESIS software. To evaluate the Hi-C assembly results, for Hi-C data assembled into chromosome, the genome was divided into 100 kb of sequence per bin, and the number of Hi-C read pairs between any two bins was calculated based on the intensity signal of the interaction between the two bins. These values were used to construct a heatmap.

### 2.4. Genome Annotation

De novo prediction of gene coding sequences was performed using Genscan [19], Augustus v2.4 [20], Glimmer HMM v3.0.4 [21], and GeneID v1.4 [22]. GeMoMa v1. 3.1 [23] was used for homologous species-based prediction. Hisat v2.0.4 [24] and Stringtie v1.2.3 [25] were used for assembly based on the reference transcripts, and TransDecoder v2.0 (https://help.rc.ufl.edu/doc/UFRC_Help_and_Documentation) and GeneMarkS-T V5.1 [26] were used for gene prediction. PASA v2.0.2 was used to predict unigene sequences based on transcriptome data without the reference assembly. Finally, EVM v1.1.1 was used to integrate the prediction results obtained by the three methods described above, and PASA v2.0.2 was used to modify the final gene models. The raw transcriptome data were compared with the *L. japonicus* genome with TopHat [27], and the number of bases in exon, intron, and intergenic regions was determined statistically to evaluate the gene prediction results.

The Rfam database and the miRBase [28] database with Infenal 1.1 were used to predict rRNAs and microRNAs, and tRNAs were identified using tRNAscan-SE v1.3.1 [29]. The pseudogene has a sequence similar to a functional gene, but it has lost its original function due to mutations such as insertions and deletions. GenBlastA v1.0.4 [30] alignment was used to search for homologous gene sequences (possible genes) in the genome of the masked whole locus, and GeneWise v2.4.1 [31] was used to find premature stop codons and frameshift mutations to obtain pseudogenes. For repeat sequence annotation, we used LTR FINDER v1.05 [32], MITE-Hunter [33], RepeatScout v1.0.5 [34], and PILER-DF v2.4 [35] to build a database of repetitive *Lotus japonicus* genome sequences based on structural prediction and de novo prediction. PASTEClassifier [36] was used to classify the database, which was then merged with the Repbase [37] database as the final repetitive sequence database. RepeatMasker v4.0.6 [38] software was used to predict repeat sequences based on the newly generated database.

To functionally annotate the genes, the predicted gene sequences were compared by BLAST v2.2.31 (e-value 1.0 × 10^−5^) with the NR [39], KOG [40], GO [41], KEGG [29], and TrEMBL [30] databases. KEGG was used for pathway analysis, and KOG and GO were used for functional analysis. InterProScan v5.8-49.0 [42] software was used to align the amino acid sequences of the predicted genes to the PROSITE [43], HAMAP [44], Pfam [45], PRINTS [46], ProDom [47], SMART [48], TIGRFAMs [49], PIRSF [50], SUPERFAMILY [51], CATH-Gene3D [52], and PANTHER [53] databases to identify and annotate the predicted motifs.

### 2.5. Genome Alignment and Gene Synteny Analysis

Information about the genomes was obtained from the Ensembl Plant Database (http://plants.ensembl.org/index.html), Ensembl Bacteria Database (https://bacteria.ensembl.org/index.html), and Phytozome v12.1 Database (https://phytozome.jgi.doe.gov/pz/portal.html). The duplication events were identified and synteny analysis was performed using MCScanX [54], JCVI (https://devhub.io/repos/tanghaibao-jcvi), and Circos (http://circos.ca/).

### 2.6. Phylogenetic Analysis

OrthoFinder (https://github.com/davidemms/OrthoFinder) [55] software was used for phylogenetic analysis of 10 Leguminosae species. OrthoFinder finds orthogroups and orthologs, generates rooted gene trees for all orthogroups, and identifies all gene duplication events in these gene trees. Orthofinder also generates a rooted species tree for the species being analyzed and maps the gene duplication events from the gene trees to branches in the species tree. Finally, OrthoFinder provides comprehensive statistics for comparative genomic analyses. Divergence times were estimated using r8s (http://hydrodictyon.eeb.uconn.edu/eebedia/index.php/Phylogenetics:_r8s_Lab#Using_r8s_to_estimate_divergence_times). The species tree was drawn using FigTree (http://tree.bio.ed.ac.uk/publications/491/).

## 3. Results

### 3.1. Genome Sequencing and Assembly 

Using third-generation sequencing technology, the original sequencing reads appeared as dumbbell-shaped structures containing junctions at both ends, as revealed by comparative physical mapping. The genomic DNA of *L. japonicus* was interrupted using g-TUBE followed by connecting with a dumbbell-shaped connector before construction of a library for sequencing. BluePippin was used to screen the target DNA fragments for the creation of sequencing libraries. Qubit2.0 and Agilent 2100 were used to detect the library concentration and insert size, respectively. The concentration of the library was further quantified using the Q-PCR method. After high-throughput sequencing (sequencing depth is about 60×), sequencing data are evaluated and filtered to obtain high-quality data (subreads) of 43.57 Gb with more than 97.43% of Q20 ratio and more than 93.81% of Q30 ratio, respectively. The mean read length was 9328 bp (Table 1). The resulting set of sub-reads contained 4,671,067 reads, with an average length of >9.32 kb. All the reads were assembled using Canu software and corrected using Pilon software based on second-generation data. After assembly, 593 contigs were obtained, with a total length of 544,144,611 bp. The contig N50 was 2,811,151 bp, and the contig N90 was 709,636 bp. The longest contig was 11,738,399 bp. The GC content was ~37.99%.

We obtained 28.06 Gb of clean data using Hi-C (high-throughput chromatin conformation capture) sequencing. The sequencing coverage was 51× and the Q30 ratio reached 92.66%. After Hi-C assembly, LACHESIS software was used to divide, rank, and orient the genomic sequence into groups for evaluation of the assembly results. A total of 517.54 Mb of the genomic sequences were mapped to the six chromosomes of *L. japonicus*, accounting for 95.1% of the total. Among these sequences, the sequence length capable of determining order and direction is 499.06 Mb, accounting for 96.43% of the total length of the mapped chromosome. The corresponding number of sequences was 537, and the total sequences clustered with a percentage of 87.18%.

### 3.2. Assessment of the Genomic Assembly

We evaluated the assembly results using three methods: (1) we compared the assembly to the second-generation sequencing data, (2) we calculated the single-base error rate, and (3) we assessed genome integrity. When we compared the assembly to the second-generation sequencing data, we obtained 222,255,924 clean reads, indicating that 98.93% of the sequences could be mapped to the reference genome. The paired-end sequences were mapped to the reference genome. The distance between sequences is consistent with the length distribution of the sequenced fragments (~96.96%). Analysis of the single-base error rate revealed that 252 bp were inconsistent, comprising 0.000046% of the total length of contigs, indicating that the single-base error rate was approximately 0.000046%.

**Figure 1:**
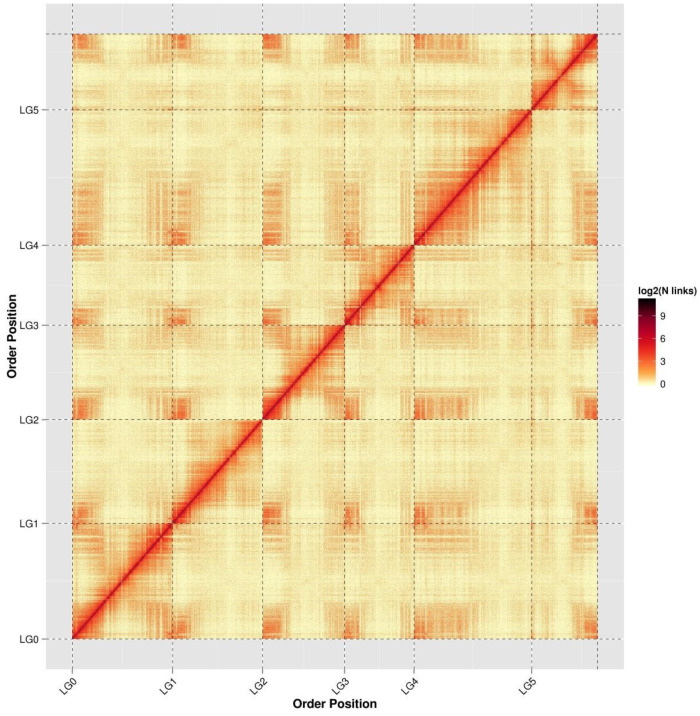
Hi-C assembly chromosome interaction heat map. LG0–LG5 represent LACHESIS groups 0–5. The abscissa and ordinate represent the order of each bin on the corresponding chromosome group.

Hi-C is an extension of chromosome conformation capture (3C) technology. An evaluation of the Hi-C assembly results showed that the six chromosome groups could be clearly distinguished. Within each group, the intensity of the interaction at the diagonal position (Figure 1) is higher than that of the non-diagonal position, indicating a high intensity of interaction between adjacent sequences (diagonal position) and a weak interaction signal between non-adjacent sequences (non-diagonal positions), which is consistent with the principle of Hi-C genome assembly.

### 3.3. Genome Annotation

**Figure 2:**
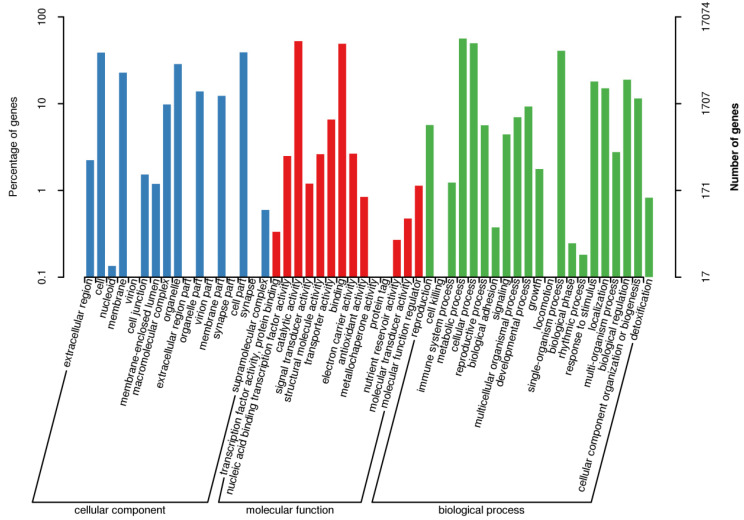
Gene ontology annotation secondary node classification statistics. The abscissa shows the content of each GO category. The left panel of the ordinate shows the percentage of each type of gene, and the right panel shows the number of genes. This figure shows the enrichment of genes for each secondary GO function.

Based on the newly constructed repetitive sequence database, we predicted the number of repeat sequences in *L. japonicus*. There were approximately 357 Mb of repetitive sequences, accounting for 65.61% of the genome. We predicted the genetic structure of *L. japonicus* using de novo prediction, homologous species prediction, and unigene prediction. We then used EVM v1.1.1 software to integrate the prediction results and PASA v2.0.2. to modify the final gene models. We ultimately obtained 28,251 genes (Table S1). Non-coding RNA prediction and pseudo-gene annotation statistics are shown in Table S1. Motif annotation revealed 2788 motifs and 33,544 domains. Based on the predicted genes, a total of 27,991 genes could be annotated to databases such as NR (Table S2). In total, 14,794 predicted genes were annotated to the KOG database, accounting for 52.37% of the total (Figure S3). Finally, 17,074 predicted genes were annotated to the GO database, accounting for 60.44% of the total (Figure 2).

The embedophyta_odb9 database in BUSCO v2 contains 1440 conserved core genes in the terrestrial plants. We used BUSCO to assess genome integrity. Among the genes used, 1335 complete BUSCO genes were identified, 1244 of which were single-copy genes. Only 75 genes were not found in the embryophyta_odb9 library. The BUSCO genome integrity score was 92.71%. The CEGMA assessment was further performed to identify 448 of the 458 core genes (97.82%) in the *L. japonicus* genome and 227 of the 248 highly conserved sequences (91.53%) in the *L. japonicus* genome. The quality of Hi-C libraries was confirmed by comparing efficiency, insert length, and effective Hi-C data volume. The sample reads and assembled genomes had an efficiency of 94.51%, and the ratio of unique mapped read pairs was 43.85%, indicating that they were suitable for subsequent analyses. Evaluation of insert length showed that the main peak sequence length of the inserted sample was ~350 bp. No deviation from the target region indicates that the insert size exhibits a normal distribution (Figure S2). An evaluation of the effective Hi-C data volume showed that the library obtained a unique alignment of 41.09 M of sequence to the genome, 32.69 M of which were valid Hi-C data, accounting for 79.56% of the data for the genome.

### 3.4. Synteny and Species Evolutionary Analysis

**Figure 3:**
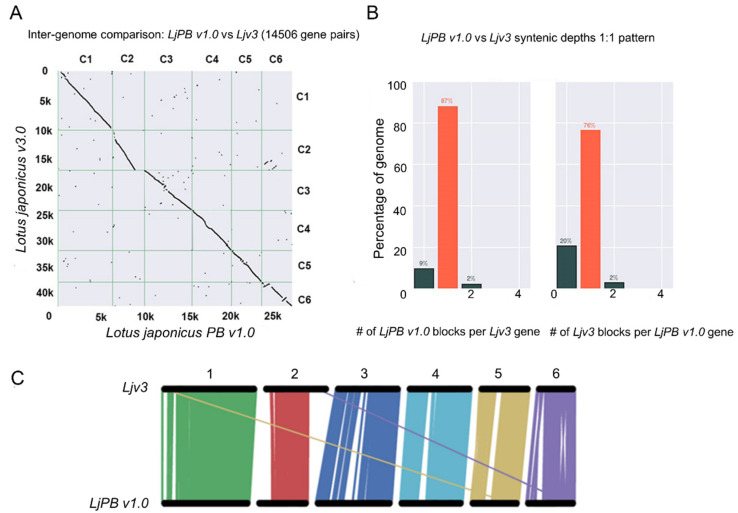
Inter-genome comparison. (**A**) Dotplot to visualize pairwise synteny. (**B**) Confirmation of the 1:1 synteny pattern. (**C**) Comparative physical mapping between *LjPB_ver1.0* and *Ljv3* shows similarity matching and mismatching of chromosome fragments.

We compared all predicted protein sequences with the published data to indirectly reveal the genome structure. A comparison of genomic structures revealed a high level of synteny between our newly generated *LjPB_ver1.0* sequence and the previously published *L. japonicus* v3.0 (MG-20). The chromosome structures of both genomes were almost identical except for a few fragments. For 87% of the sequences, we found the corresponding block in *LjPB_ver1.0*, and for 2% of the sequences, we found two corresponding blocks in *LjPB_ver1.0*. For 9% of the *L. japonicus* v3.0 (MG-20) protein sequences, we could not find the corresponding block in *LjPB_ver1.0*. By contrast, for 20% of *LjPB_ver1.0* sequences, we could not find the corresponding block in *L. japonicus* v3.0 (MG-20). For 76% of these sequences, we found the corresponding block in *L. japonicus* v3.0 (MG-20), and for 2% of the sequences, we found two corresponding blocks in *L. japonicus* v3.0 (MG-20). Both genomes contain ~2% of sequences with a lower degree of matching; we therefore did not include these sequences in our statistical analysis (Figure 3).

**Figure 4:**
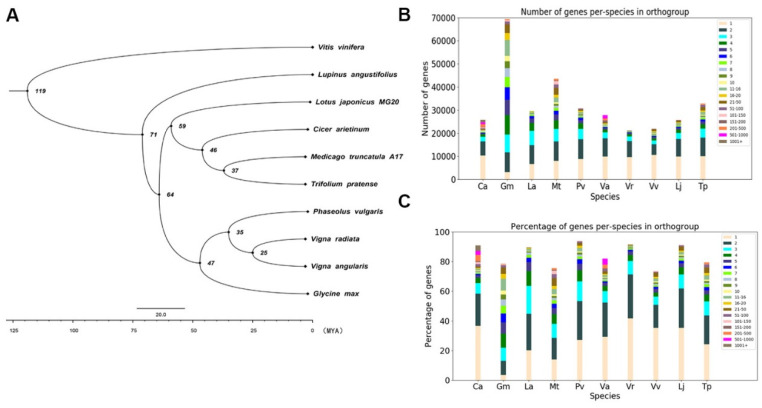
Species phylogenetic trees and statistics of orthologous genes among species. (**A**) genome-wide phylogenetic tree of nine legume species, showing their phylogenetic relationship and time of divergence. (**B**) Statistical analysis of orthologous genes between species. Different colors indicate the number of different orthologous genes, and the histogram represents the total number of different orthologous genes of a single species. (**C**) comparison of the proportions of different orthologous genes in the genomes of a single species.

We constructed a phylogenetic tree based on sequence alignment of single-copy gene families shared by *Vitis vinifera*, *Lupinus angustifolius*, *L. japonicus* (MG-20), *Cicer arietinum*, *Medicago truncatula* (A17), *Trifolium pratense*, *Phaseolus vulgaris*, *Vigna radiata*, *Vigna angularis*, and *Glycine max*. We calculated the divergence times of various species with r8s software (expressed in millions of years ago [MYA]) (Figure 4). Based on an orthogroup analysis, we showed statistics concerning the orthologous genes among different species. The legend indicates the number of different orthologous genes, and the histogram represents the total statistics of the number of different orthologous genes of a single species. The other histogram shows the proportion of different orthologous genes in the genome of a species. The total proportion of orthologous genes in each species is not 100%, because, for some of the genes, we did not find orthologs in other species, perhaps because they are species-specific genes (Figure 4).

**Figure 5:**
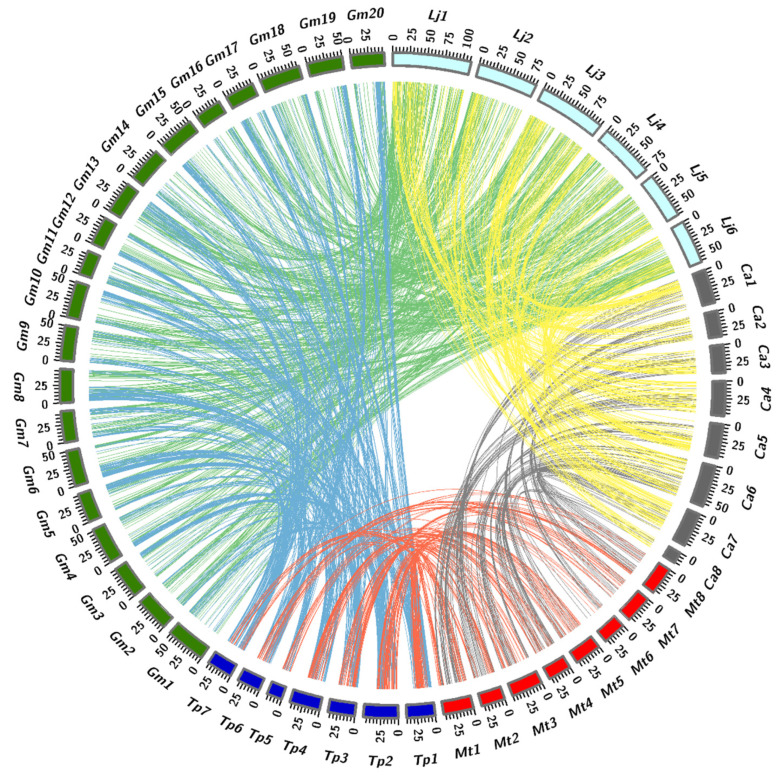
Circos diagram illustrating syntentic relationships between *Medicago, Glycine, Lotus, Cicer* and *Trifolium*. Based on phylogenetic analysis comparing the synteny of adjacent species on the phylogenetic tree branch, which show a high degree of synteny.

**Figure 6:**
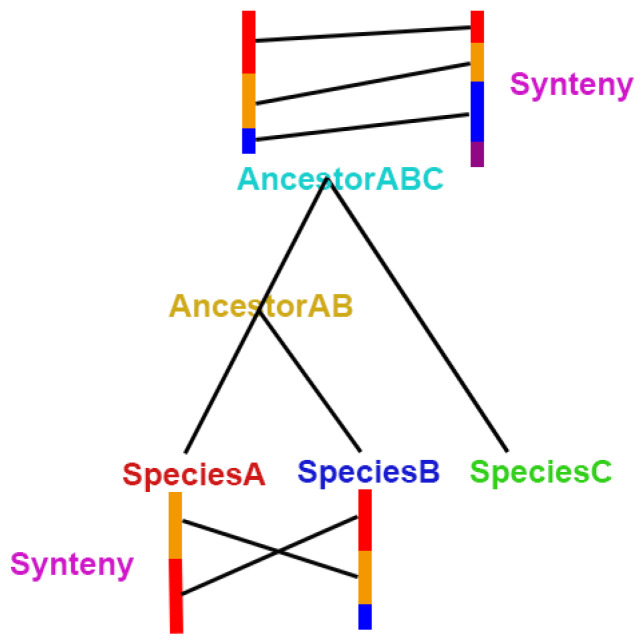
Example of a pattern diagram for inferring the chromosomes from ancestral genomes based on synteny analysis.

Synteny analysis of the *L. japonicus* genome with the genomes of other legume species revealed strong synteny between species. Although the *L. japonicus* genome only has six chromosomes, one of the chromosomes is longer than the others. Synteny analysis also indicated that the chromosomal fragments containing the most genes had a large number of corresponding regions in the genomes of other species. Perhaps during evolution, chromosome breaks or fusions occurred that led to the formation of different legume species (Figure S4). Based on the results of phylogenetic analysis, a comparison of the synteny of adjacent species on a phylogenetic tree branch revealed a high degree of synteny. Information obtained from a comparison of synteny between neighboring species in the evolutionary tree at the genome level might be used to infer the general chromosomes of their ancestor species, which could shed light on the origin of nodulation in legumes (Figure 5). We constructed a pattern diagram based on this idea (Figure 6). Based on the results of phylogenetic analysis, we compared the synteny of adjacent species on a phylogenetic tree branch. We proposed a possible chromosomal rearrangement based on the linear correspondence of the chromosome fragments of the comparison results, which might have occurred in the ancestral species of the A and B species. Alternatively, perhaps the A and B species are closely related and do not share an intermediate ancestor species, but the A species might have arisen as a new species due to a chromosomal mutation in the B species. Based on our subjective analysis of phenotypes, species with better phenotypes are considered to be evolutionarily successful species, and species with weaker phenotypes are considered to be ancestral species.

### 3.5. Orthogroup Analysis

**Figure 7:**
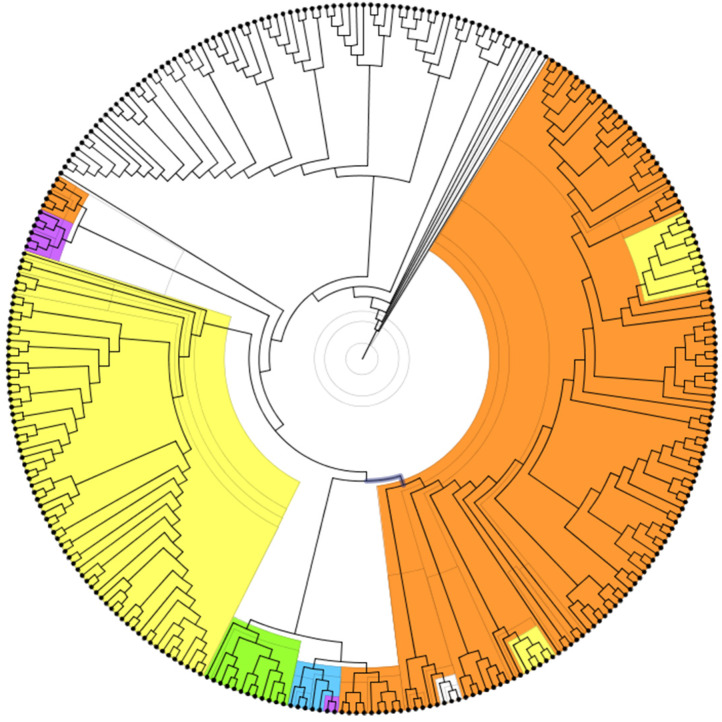
Phylogenetic tree of the largest gene family in *L. japonicus* and the orthologous genes of several other legume species. genes from *L. japonicus* are shown in orange, genes from *P. vulgaris* are shown in yellow, genes from *G. max* are shown in white, genes from *L. angustifolius* are shown in purple, genes from *M. truncatula* (A17) are shown in green, while genes from *T. pratense* are shown in blue.

We performed orthogroup analysis of the ten plant species and identified 5436 orthogroups that were related to *LjPB_ver1.0* (Table S3, Table S4). We focused on the largest orthogroup in *LjPB_ver1.0*, which contains 96 genes. Among the orthologous genes of other species, there are 82 orthologous genes in *G. max*, 11 in *L. angustifolius*, 11 in *M. truncatula* (A17), 86 in *P. vulgaris*, and 6 in *T. pratense*. No homologous genes are present in *V. vinifera*, *C. arietinum*, *V. radiata* or *V. angularis* (Figure 7). The total number of genes in this orthogroup is 292. However, surprisingly, the functions of all of these genes are unknown, and the proteins have not been characterized (Table S5).

**Figure 8:**
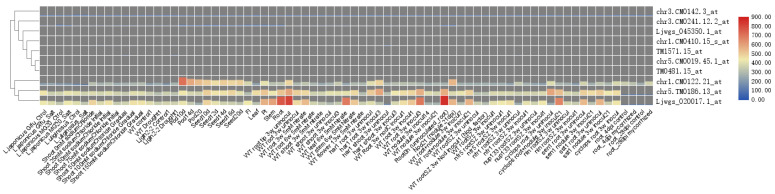
The expression levels of 10 gene probes in this gene family indicate that some genes have higher expression levels in roots, stems and pods, and perhaps the entire gene family plays an important role in plant growth and development.

We checked the expression data for these genes in the Noble Research Institute website (https://ljgea.noble.org/v2/index.php). Among these 96 genes, we found the expression levels of only 12 genes, some of which are highly expressed in roots, stems, and pods (Figure 8). These findings suggest that these genes play important roles in plant growth and development in some legume plants. They also highlight the need to further study this huge orthogroup. We also checked the same information on the LotusBase website (https://lotus.au.dk/) [56,57,58,59]. Based on Blastp, we set “-max_target_seqs 1” to ensure that the *LjPB_ver1.0* gene and Ljv3 expression data correspond to each other. However, as shown in Table S6, there are some differences between the two genomes. After removing the repeated IDs from all the gene IDs obtained, 37 genes were identified. Among the 37 genes, only five genes were identified to have expression data in the LotusBase (Figure S1). The difference between the two genomes also indicates that the accuracy of sequencing and assembly is subject to verification by biological experiments. In fact, while it is difficult to verify the functions of such a large group of orthologous genes, further analysis of the orthogroup revealed that this orthogroup has only a few members in *M. truncatula* (A17), *L. angustifolius*, and *T. pratense*. The data suggest that we can study the functions of these genes in these three species to infer the possible functions of the largest group of orthologous genes in *L. japonicus* through functional annotation.

## 4. Discussion

In the current study, we generated a high-quality genome sequence for the model legume *L. japonicus*. This sequence contains a large amount sequence information, high sequencing depth, and long contigs. The high quality of our genome assembly makes it an excellent platform for molecular biology and genetic research. An analysis of orthologous genes in the whole genomes of several leguminous plants will provide insight into species evolution caused by the formation of gene families and gene clusters via repeats and mutation events. However, this analysis must be performed using two species that are particularly close to each other before they can be used to infer ancestral chromosome composition. Otherwise, there may be multiple mutations in chromosome compositions due to the large number of intermediate species. Such complexity could be confusing when trying to obtain information about gene synteny, preventing researchers from making accurate inferences. Therefore, studies on the evolution of species still require more genome-wide sequencing data.

## 5. Conclusions

Comparative genomics studies using bioinformatics techniques revealed similarities and differences between the re-sequenced and assembled genomes and previously published versions. Based on assembly evaluation and annotation of gene function evaluation, our data has higher accuracy, which will provide researchers with more accurate genomic information. We have submitted the data to Phytozome, which will soon be published on the website for use. The identification of all orthologous genes in 10 species will help provide critical evolutionary information when studying genes’ functions.

## 6. Patents

### Availability of Supporting Data and Materials 

Some codes and original files for comparative genome analysis have been uploaded to Github (https://github.com/LHXqwq/A-High-quality-Genome-Sequence-of-Model-Legume-Lotus-japonicus-MG-20-provides-insights-into-the-ev.git).

## Figures and Tables

**Table 1:** Summary of genome assembly and comparison with previous assembly *L.j v3.0.*

Genomic Feature	*LjPB_ver1.0*	*L. japonicus (MG-20) v3.0*
Total length of contigs	544,144,611	NA
Total sequences length of clustered	517,538,720	NA
Total sequences ordered and oriented	499,061,371	394,454,697
Percentage of total sequences clustered	95.11%	NA
Percentage of total sequences ordered and oriented	96.43%	NA
Number of contigs	616	44,464
Contig N50(bp)	2,515,607	25,054
Contig N90 (bp)	675,000	NA
Contig max (bp)	11,738,399	NA
GC content	37.99%	36.6%
Genome coverage	60×	35×
Percentage of repeat sequences	65.61%	NA
Percentage of 458 CEGs present in assemblies	97.82%	NA
Percentage of 248 highly conserved CEGs present	91.53%	NA
Number of genes	28,251	10,951

## Supplementary Materials

The following are available online at https://www.mdpi.com/2073-4425/11/5/483/s1, Supplementary Table S1: Gene prediction result statistics. Gene information statistics. Non-coding RNA statistics. Pseudogene prediction results. Supplementary Table S2: Gene function annotation statistics. Supplementary Table S3: Statistics the number of orthologous genes in various species. Supplementary Table S4: Orthologous gene id statistics of various species. Supplementary Table S5: Annotation of the largest orthologous in L. japonicus (MG-20). Supplementary Table S6: The blastp results of the largest orthologous gene family of L. japonicus (MG-20). Supplementary Figure S1: The largest gene family of L. japonicus found only the expression data corresponding to 5 genes in LotusBase. Supplementary Figure S2: Map of library insert length. Supplementary Figure S3: KOG functional annotation classification chart. Supplementary Figure S4: Synteny analysis of the *LjPB_ver1.0* genome with the genomes of other legume species.
