# Supplementary material for: A High-Quality Genome Sequence of Model Legume Lotus japonicus (MG-20) Provides Insights into the Evolution of Root Nodule Symbiosis
Source: Genes (Basel). 2020 Apr 29;11(5):483. doi: 10.3390/genes11050483 (PMC7290416; doi:10.3390/genes11050483)
Supplement: Supplementary file 1 [file genes-11-00483-s001.zip › Supplementary Figs.docx]

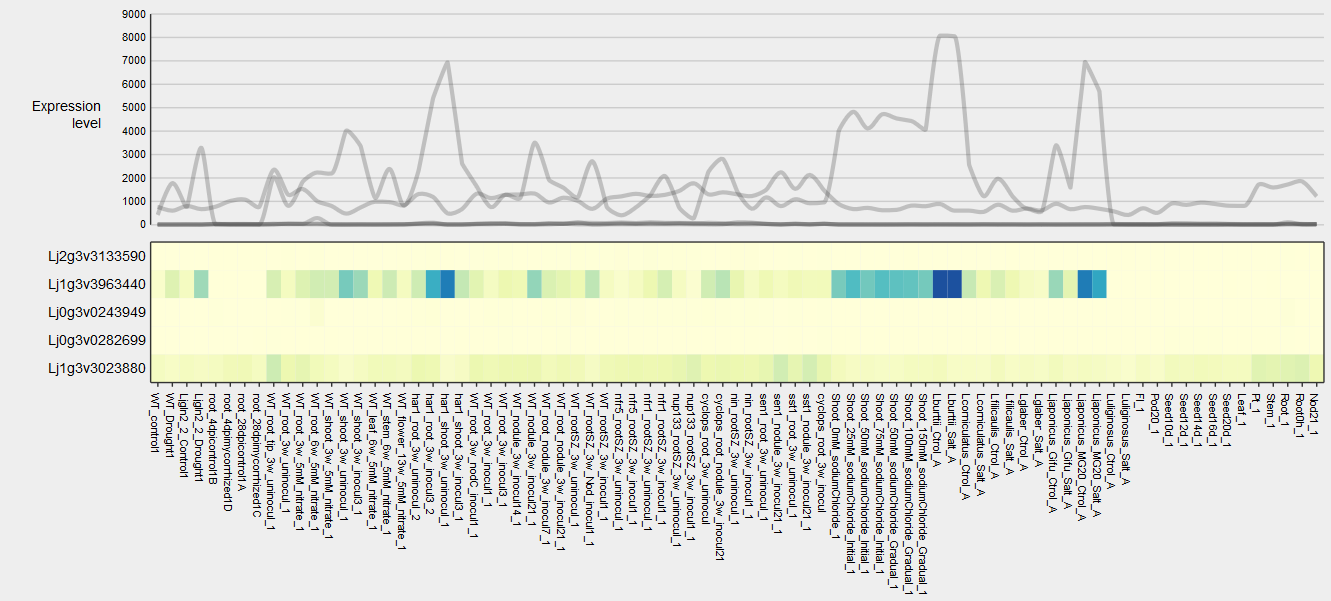


**Supplementary Figure 1:** The largest gene family of *L. japonicus* found only the expression data corresponding to 5 genes in LotusBase. Among these data, the Lj1g3v3963440 gene was found to be highly expressed in shoots, roots, and flowers. The exact function of the gene is not yet known, but it has been predicted to contain RNA-binding region RNP-1 (RNA recognition motif). Phylogenetic analysis shows that it has a large number of orthologous gene members. Whether other members perform the same function, and it needs biological data to prove that it may be possible to construct mutant materials from fewer orthologous genes in other species to explore potential features.

**
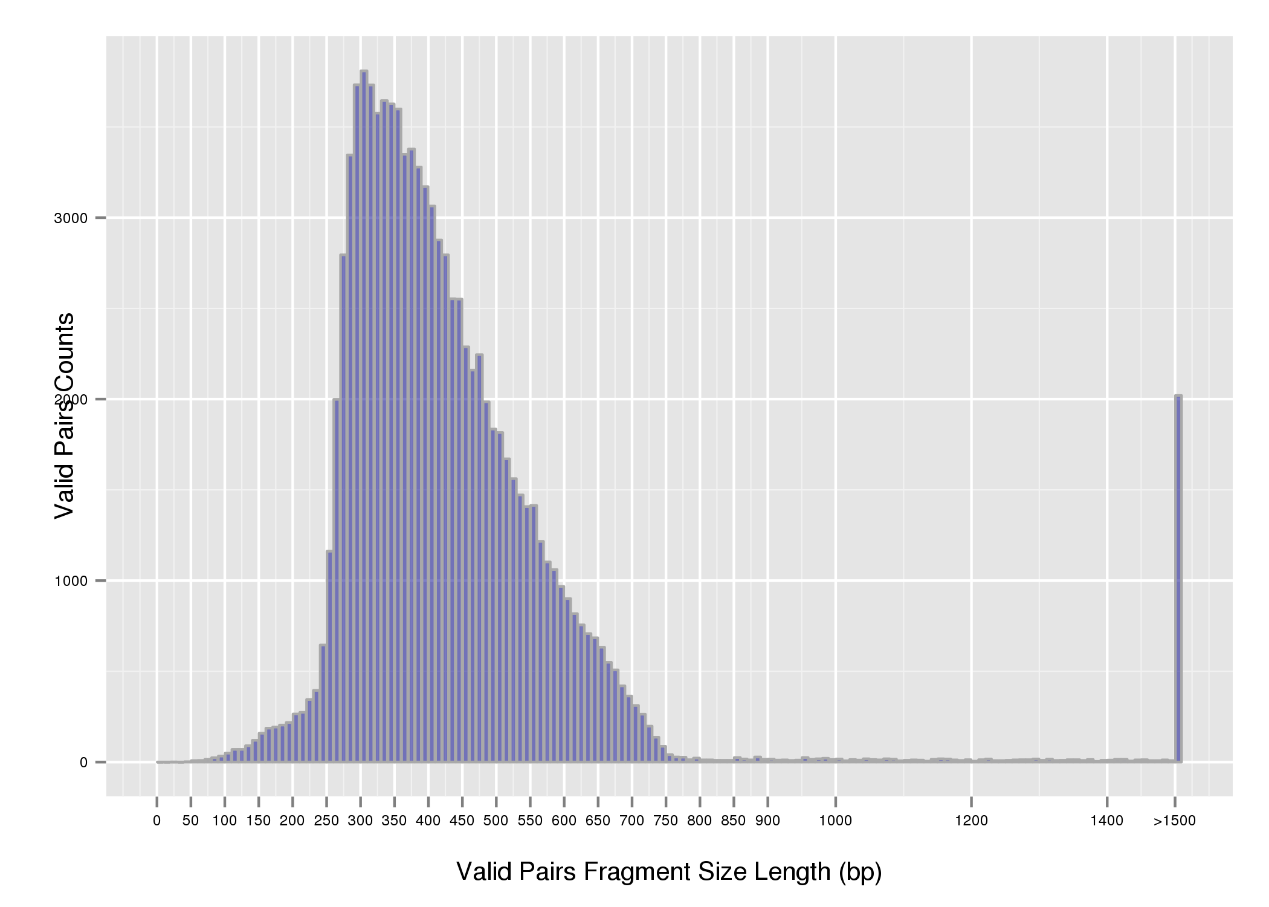
**

**Supplementary Figure 2:** Map of library insert length. The abscissa shows the sum of the distances between the paired-ended reads of assembled genomes and the nearest Hind III restriction sites. The ordinate shows the number of randomly selected 100,000 pairs of reads at different lengths of the insert.

**
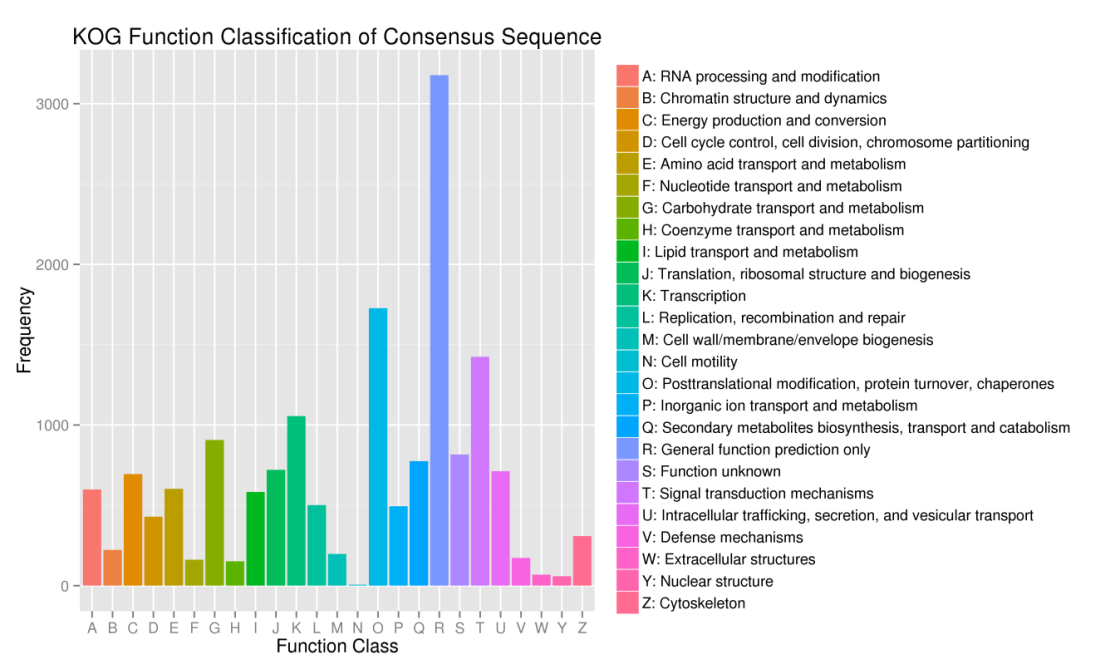
**

**Supplementary Figure 3:** KOG functional annotation classification chart. The abscissa represents the content of each KOG category, and the ordinate shows the number of genes.


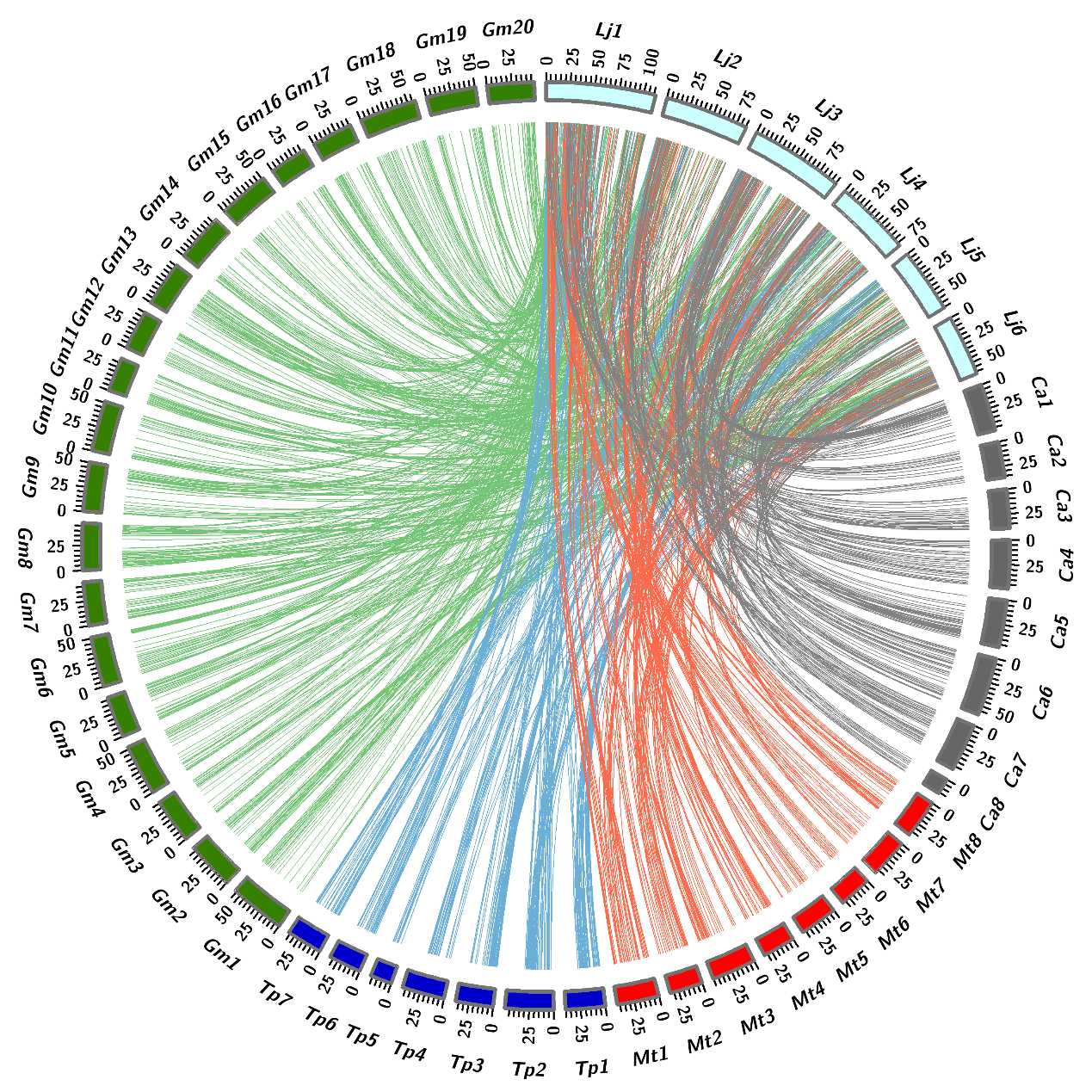


**Supplementary Figure 4:** Synteny analysis of the *LjPB_ver1.0* genome with the genomes of other legume species.
